# Isolated central nervous system relapse in mantle cell lymphoma

**DOI:** 10.1002/jha2.1081

**Published:** 2025-01-06

**Authors:** Radu Chiriac, Zofia Gross

**Affiliations:** ^1^ Hematology Laboratory Hospices Civils de Lyon Centre Hospitalier Lyon Sud Lyon France; ^2^ Department of Hematology Hospices Civils de Lyon Centre Hospitalier Lyon Sud Lyon France

**Keywords:** CNS, cytology, MCL, relapse

1

A 62‐year‐old male presented with a 3‐week history of back pain, fatigue, and progressively enlarging lymph nodes in the inguinal region. Initial blood tests revealed anemia (78 g/L), leukocytosis (88 × 10^9^/L), and elevated lactate dehydrogenase (LDH) (1400 U/L).

An incisional biopsy of the inguinal lymph node confirmed the diagnosis of the blastoid variant of mantle cell lymphoma (MCL), characterized by a very high proliferation rate (Ki‐67: 90%). Imaging revealed extensive lymphadenopathy both above and below the diaphragm, along with 80% bone marrow involvement. High‐risk features at diagnosis included stage IVB disease, a high‐risk biological Mantle Cell Lymphoma International Prognostic Index score of 9, and skeletal involvement. No cerebrospinal fluid (CSF) infiltration was observed at the time of diagnosis.

The initial treatment consisted of four cycles of rituximab, dexamethasone, cytarabine, oxaliplatin (R‐DHAOx). Interim positron emission tomography‐computed tomography after three cycles of R‐DHAOx showed significant improvement in lymphadenopathy and skeletal lesions, with no new lesions. Due to persistent cholestasis the planned autologous stem cell transplantation (aHSCT) was postponed, and two cycles of R‐DHA were administered to minimize hepatotoxicity.

However, 7 months after the initial presentation (while in remission), the patient presented with a decline in overall condition, including 10 days of night sweats, balance disturbances with ataxic gait, lower limb weakness, and hypoesthesia. Magnetic resonance imaging (MRI) confirmed the presence of an intracanalar spinal lesion at the T5‐T6 vertebral level, located posteriorly in the extradural space, measuring 50 × 10 mm (Figure 1A, asterisk).

**FIGURE 1:**
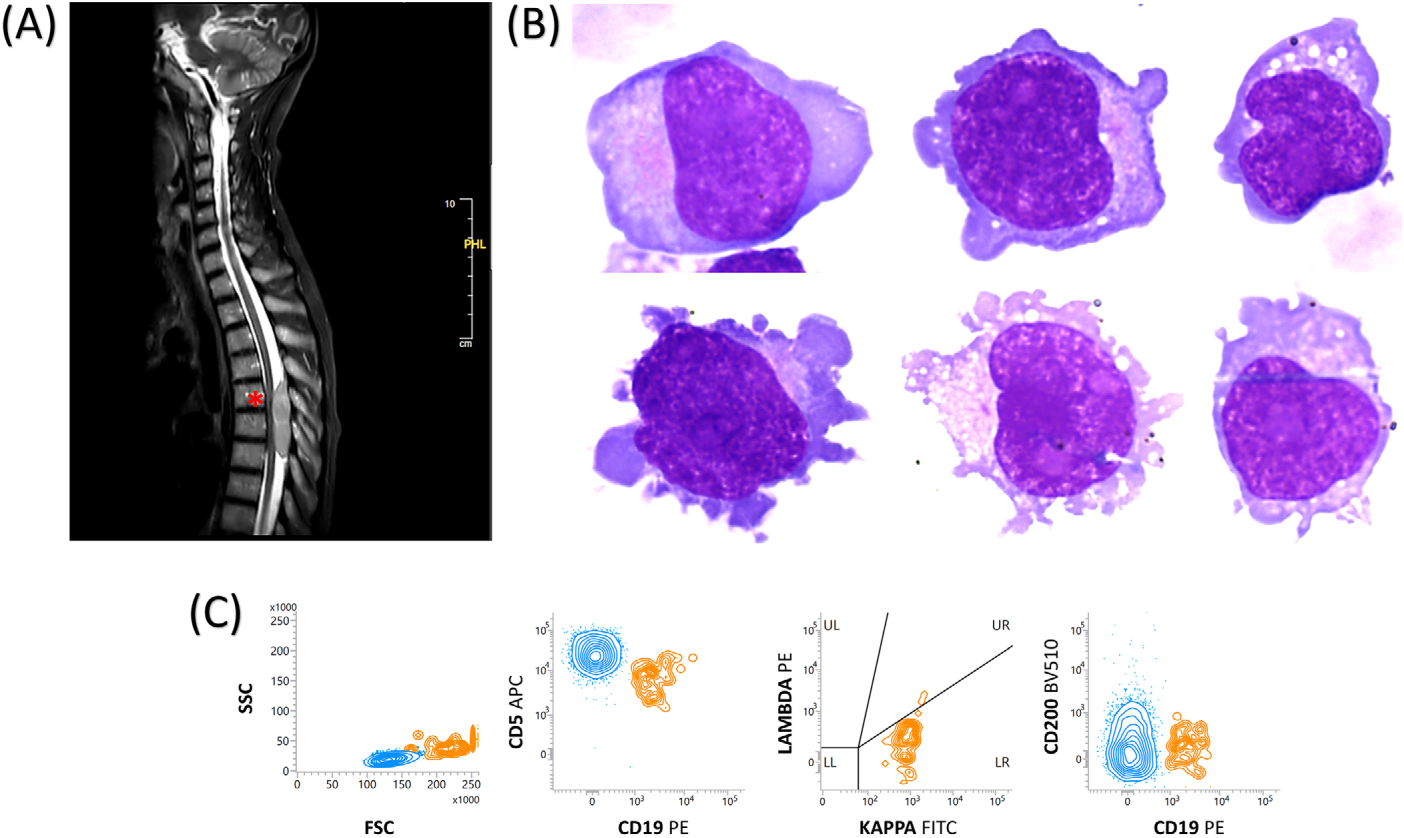
Panel (A) T2‐weighted turbo spin echo magnetic resonance imaging (MRI) sagittal sequences reveal the presence of an intracanalar spinal lesion at the T5‐T6 vertebral level, located posteriorly in the extradural space, measuring 50 × 10 mm (asterisk). Panel (B) Cerebrospinal fluid cytospin preparation, May‐Grünwald Giemsa stain, ×100 objective, revealing monomorphic, medium‐sized lymphomatous cells exhibiting regular nuclear contours, variably condensed nucleolated chromatin, and scant to moderate cytoplasm. Panel (C) Flow cytometry results showing a kappa‐restricted B‐cell population with the following markers: CD19+, CD5+, and CD200‐ (orange population).

A lumbar puncture revealed a CSF cytospin preparation with monomorphic, medium‐sized lymphomatous cells exhibiting regular nuclear contours, variably condensed nucleolated chromatin, and scant to moderate cytoplasm (Figure 1B). Flow cytometry of the CSF confirmed a large monoclonal kappa B‐cell population: CD5+, CD19+, and CD200‐negative (Figure 1C), confirming a central nervous system (CNS) relapse of blastoid MCL. No circulating lymphoma cells were detected.

The patient initially received high‐dose corticosteroids for 4 days, which resulted in a slight improvement in neurological symptoms. Following this, the first cycle of rituximab, methotrexate, procarbazine, and vincristine (R‐MPV) was initiated, alongside the start of ibrutinib. Three weeks later, the patient experienced severe headaches, paroxysmal vertigo, and confusion. MRI showed a reduction in the intracanalar lesion, but cytology confirmed the presence of residual lymphomatous infiltration. In response, a revised treatment regimen comprising rituximab, bendamustine, and continued ibrutinib was instituted, yielding sustained clinical improvement with no evidence of relapse to date. Plans for aHSCT are currently in progress.

This case highlights the complexities of managing high‐risk MCL with CNS involvement. Despite initial challenges, the patient demonstrated a positive response to a revised treatment regimen, leading to sustained improvement and stabilization of neurological symptoms.

CNS involvement in MCL is rare, challenging to treat, and associated with poor outcomes, including short overall and progression‐free survival [1]. Advanced stage, blastoid variant, elevated LDH, and high Ki67 are more common at diagnosis in CNS MCL cases [2]. Furthermore, first‐line CD19‐based chimeric antigen receptor T‐cell monotherapy may offer a viable option for patients unsuitable for intensive chemotherapy and could be preferable to ibrutinib due to its superior toxicity profile [3].

## AUTHOR CONTRIBUTIONS

Radu Chiriac wrote the manuscript and conducted the cytologic studies. Zofia Gross followed the patient and provided patient information. All authors contributed to the final manuscript.

## CONFLICT OF INTEREST STATEMENT

The authors have no conflict of interest.

## FUNDING INFORMATION

The authors did not receive support from any organization for the submitted work.

## ETHICS STATEMENT

This manuscript respects the ethics policy of CHU Lyon for the treatment of human research participants.

## PATIENT CONSENT STATEMENT

No patient‐identifying data were used. The authors did not obtain written informed consent from the patient but the patient did not object to his data being used for research purposes (as required by the ethics policy of CHU Lyon).

## CLINICAL TRIAL REGISTRATION

Not applicable.

## Data Availability

Data sharing is not applicable to this article as no new data were created or analyzed in this study.
